# Harmonization of the intracellular cytokine staining assay

**DOI:** 10.1007/s00262-012-1282-9

**Published:** 2012-05-22

**Authors:** Marij J. P. Welters, Cécile Gouttefangeas, Tamara H. Ramwadhdoebe, Anne Letsch, Christian H. Ottensmeier, Cedrik M. Britten, Sjoerd H. van der Burg

**Affiliations:** 1 Department of Clinical Oncology, Building 1, K1-P, Leiden University Medical Center, PO box 9600, 2300 RC Leiden, The Netherlands; 2 Department of Immunology, Interfaculty Institute for Cell Biology, Eberhard Karls University, Tübingen, Germany; 3 Department of Hematology, Oncology, Charité Campus Benjamin Franklin, Berlin, Germany; 4 Cancer Sciences Division, Southampton University Hospitals, Southampton, UK; 5 TRON—Translational Oncology, University Medical Center of Johannes Gutenberg University, Mainz, Germany

**Keywords:** T cells, Intracellular cytokine staining, Flow cytometry, Proficiency panel, Harmonization

## Abstract

Active immunotherapy for cancer is an accepted treatment modality aiming to reinforce the T-cell response to cancer. T-cell reactivity is measured by various assays and used to guide the clinical development of immunotherapeutics. However, data obtained across different institutions may vary substantially making comparative conclusions difficult. The Cancer Immunotherapy Immunoguiding Program organizes proficiency panels to identify key parameters influencing the outcome of commonly used T-cell assays followed by harmonization. Our successes with IFNγ-ELISPOT and peptide HLA multimer analysis have led to the current study on intracellular cytokine staining (ICS). We report the results of three successive panels evaluating this assay. At the beginning, 3 out of 9 participants (33 %) were able to detect >6 out of 8 known virus-specific T-cell responses in peripheral blood of healthy individuals. This increased to 50 % of the laboratories in the second phase. The reported percentages of cytokine-producing T cells by the different laboratories were highly variable with coefficients of variation well over 60 %. Variability could partially be explained by protocol-related differences in background cytokine production leading to sub-optimal signal-to-noise ratios. The large number of protocol variables prohibited identification of prime guidelines to harmonize the assays. In addition, the gating strategy used to identify reactive T cells had a major impact on assay outcome. Subsequent harmonization of the gating strategy considerably reduced the variability within the group of participants. In conclusion, we propose that first basic guidelines should be applied for gating in ICS experiments before harmonizing assay protocol variables.

**Electronic supplementary material:**

The online version of this article (doi:10.1007/s00262-012-1282-9) contains supplementary material, which is available to authorized users.

## Introduction

The immune system is an important component in controlling cancer development. Tumor-specific T cells make a major contribution to this effect. Immunosuppressed individuals, such as transplant recipients, have a substantially elevated risk of developing malignancy [1], and there exists a strong association between an intratumoral T-cell infiltrate and increased overall survival in many types of cancer [2]. Reinforcement of the adaptive immune response in patients with cancer through immunotherapy has now developed into an accepted modality, either as standalone therapy or in combination with standard strategies such as surgery, radiotherapy and chemotherapy. Recently, two immunotherapeutics were approved by the food and drug administration (FDA). One is the vaccine Sipuleucel-T in prostate cancer and the other the anti-CTLA-4 monoclonal antibody Ipilimumab in melanoma [3, 4]. Although efficacy of immunotherapeutic intervention is a clinical parameter, there is an urgent need for biomarkers that allow the selection of patients for immunotherapy or that predict for benefit early enough to allow treatment decisions to be made. These biomarkers need to detect changes in the patient’s immune response and are likely to reflect parameters associated with the mechanism of action identified in preclinical models.

For rational development of T-cell immunotherapeutics, robust and sensitive immunological assays able to determine the quality and quantity of tumor-specific T cells are critical. The most commonly used assays are IFNγ enzyme-linked immunospot (ELISPOT), HLA multimer staining and the intracellular cytokine staining (ICS) assay. The latter two are flow cytometry based and can provide detailed information at the single cell level. Despite the fact that these assays are widely used, it has been difficult to show a direct correlation between T-cell response and clinical course in many studies. Reasons for this are the generally low number of clinical responses observed and/or incomplete immunomonitoring of therapy-induced changes of the immune system [5, 6]. Furthermore, it is also difficult to base product development on direct interpretation of published studies, as the methods vary widely between institutes and this is exacerbated by the lack of reference samples for quality control. One way to overcome this heterogeneity is to rigorously standardize assays similar to the approach taken in the HIV vaccination field [7, 8] or to use a central laboratory [9]. The CIMT Immunoguiding Program (CIP) is an European working group that has taken the approach of optimizing and harmonizing T-cell assays through iterative proficiency panels, in which laboratories measure T-cell reactivities in the same cell samples [10]. Our goal is to improve comparability of immune data generated by the participating groups. During the last 6 years, twelve proficiency panels have been conducted by CIP, following this multistep approach. Using some standardized reagents (i.e., peptides or HLA-peptide multimers), but allowing each center to use its own protocol, parameters were identified that had a major influence on assay outcome. In further iterations, participants were asked to perform the assay again, using the harmonized protocol. This straightforward approach resulted not only in improved comparability and reproducibility of T-cell assays, but also offered regular performance feedback to participants and helps in establishing specific assay benchmarks [11–13].

Here, we describe the results of a series of three consecutive ICS proficiency panels. The first two panels showed that culture medium and background staining influence assay outcome, similar to observations from the other proficiency panels [12, 13]. These panels also revealed a second level of variability resulting from differences in data analysis. A third in silico panel demonstrated that this is a key factor in ICS analysis and that harmonization at the level of data analysis is a pre-requisite to identify protocol-specific parameters influencing assay performance and ultimately to decrease variability of results generated across institutions.

## Materials and methods

Structured information is provided according to the Minimal Information About T-cell Assays Reporting Framework for human T-cell assays [14, 15].

### Samples

#### Selection and shipment of Peripheral Blood Mononuclear Cells (PBMC) samples

Buffy coats of 7 HLA-A2-positive healthy blood donors were obtained from Sanquin Blood Donor Bank in Leiden, the Netherlands. All subjects had signed an informed consent. PBMC were processed within 24 h and isolated using Ficoll density gradient centrifugation, washed with phosphate-buffered saline (PBS), resuspended in cold Fetal Calf Serum (FCS; PAA Laboratories, Pasching, Austria) and cooled on ice for 15 min. After dropwise addition in a 1:1 ratio of freezing medium, consisting of 80 % FCS and 20 % DMSO (Sigma, St Louis, MO, USA), the PBMC were cryopreserved (12.4, 10.0, 10.0, 15.3, 13.6, 15.3 and 13.0 million PBMC per ml per vial for the buffy coats 1–7, respectively) using an automated controlled rate freezer (Cryosolutions, 's-Hertogenbosch, the Netherlands) and stored in equal aliquots in a vapor-phase liquid nitrogen vessel until use. The handling and storage of the PBMC were done according to the standard operation procedures (SOPs) of the Leiden department of Oncology by trained personnel. Cryopreserved PBMC (two vials from each donor) were transported to the participants on dry ice (minimal 5 kg/box), within 4 months after PBMC isolation and subsequent storage. Samples reached the participating laboratory within 2 days after shipment and upon arrival were stored in liquid nitrogen as agreed by the panel guideline.

#### Antigens

To quantify CD8+ T-cell responses by ICS, reactivity to FLU_58–66_ (GILGFVFTL) from Influenza Matrix 1 protein and CMV pp65_495–503_ (NLVPMVATV) from human cytomegalovirus was assessed. Peptides were centrally synthesized with >95 % purity [16], dissolved in PBS with 2 % DSMO at 1 mg/ml, and 20 μl aliquots was shipped to the participants on dry ice with the cryopreserved PBMC and stored at −20 °C. PHA (HA16; Murex BioTech, Kent, UK) was taken along as a positive control in the pre-screening ELISPOT assay.

### Assays and data acquisition

#### Pre-screening to identify donors with FLU- and/or CMV-specific CD8+ T cells

Pre-screening was conducted within 1 month after cryopreservation by the central laboratory at the Leiden department of Oncology by IFNγ-ELISPOT assay according to the local SOPs, in conformity with CIP guidelines (10) (http://www.cimt.eu/workgroup/CIP). PBMC from the 7 buffy coats were thawed (using cold IMDM according to the local SOP), counted (viable and death cells by trypan blue staining), resuspended in 10 ml of IMDM (Lonza, Verviers, Belgium), 100 U/ml penicillin/100 μg/ml streptomycin (Lonza), 25 mM β-mercaptoethanol (Sigma) and 2 mM glutamine (Lonza) (i.e., complete IMDM), supplemented with 10 % heat-inactivated human AB serum (Greiner, Alphen aan den Rijn, the Netherlands). Cells (1–2 × 10^6^/ml) were rested at 37 °C, 5 % CO_2_ and 92 % overnight (18 h) in a 50-ml tube, with the cap loosened for gas exchange. The recovery of viable cells immediately after thawing ranged between 81.3 and 148.5 % (average 100 %, median 100 %, coefficient of variation (CV) value 31 %), after resting viable cells averaged at 61.1 %, median 61.6 % and CV value 22 % of input number. The ELISPOT assay was conducted in triplicate wells (500,000 c/w) according to our publicly available SOP (http://www.cimt.eu/dl/sop_elispot.pdf), except that ELISPOT plates were blocked and PBMCs resuspended (after the overnight resting phase) in complete IMDM with 10 % FCS instead of X-Vivo 15 medium. Plates were dried and measured by ELISPOT reader (BioSys 5000; software version 10.8). The settings of this ELISPOT reader were as follows: the spot size from 65 to 400, the circularity of the spot 2, the slope of the spot intensity medium and the sensitivity 81 %. A positive response had to fulfill the criteria established in CIP ELISPOT panels [12]: significantly higher spot counts in the triplicate antigen-stimulated cell samples (experimental wells) than in medium only (triplicate control wells) following a two-sided Student’s *t* test (*p* ≤ 0.05), the average spot number in the experimental wells being at least threefold that of the control wells. The ELISPOT assay was conducted twice for all 7 donors, and the results are shown in Online resource 1. For the proficiency panel, 5 donors were selected: buffy coat 1 [assigned as donor 1 (D1)] and buffy coats 4–7 [assigned as donors 2–5 (D2–D5), respectively]. A total of 8 responses were identified: 3 donors (D2, D3 and D5) responded to the HLA-A*0201-restricted CMV peptide and all 5 donors harbored T cells against the HLA-A*0201-restricted FLU peptide.

#### Design of the ICS proficiency panels

The ICS proficiency panels were conducted in a multistep (phase) approach (Online resource 2). Nine laboratories from 3 European countries (Germany, the Netherlands and UK) participated in the first panel. All laboratories were asked to determine the frequency of IFNγ-producing CD8+ T cells in the provided 5 PBMC samples (by using only one vial) with their own ICS protocol and reagents within 2 months upon sample receive. Participants reported the following: (1) thawing conditions, (2) cell recovery with or without allowing the cells to rest for a certain time and at a certain temperature (i.e., resting phase), (3) number of cells used per test condition, (4) medium (5) serum used in the test, (6) peptide concentration to stimulate the PBMC, (7) duration of activation, (8) reagent to lyse/fix the cells, (9) reagent to prevent cytokine secretion, (10) reagent to permeabilize cells, (11) antibody combination (amount, clone, company and fluorescent label), (12) duration and conditions of staining, (13) type of flow cytometer used, (14) compensation method, (15) software, (16) number of lymphocytes acquired, (17) number and percentages of CD8+ T cells acquired and finally (18) the number (and %) of IFNγ-producing CD8+ T cells upon peptide stimulation as determined by the participant (Online resource 3A and 4A). The results of this first phase were used to identify the key factors influencing the assay performance.

In the second phase, aliquots of the same PBMC samples (stored for 16 months at the participating laboratory sites) were re-tested using mandatory parameters that were deduced from the first step. Six participant laboratories reported data as in phase 1, including resting time; however, thawing conditions (point 1) and cell recovery (point 2) were not collected again (Online resource 3B and 4A). We identified that the participants’ gating strategy was a major assay variable. Therefore, in a third panel phase, all participants analyzed a set of identical flow cytometry standard (FCS) format data files 3.0, chosen from the dataset of one laboratory from panel phase 2. From each donor, the data of the non-stimulated, the FLU- and CMV-stimulated PBMC sample were provided. The participants (*n* = 10) analyzed the 9 FCS files and reported the results as described under points 16–18 (in silico ICS panel). Three laboratories undertook re-analysis of the FCS files using fixed gating instructions to harmonize the outcome.

### Data analysis and interpretation

Central analysis of the ICS panels at the Leiden department of Oncology used the numerical data reported by the participants. In the first phase, background staining (i.e., percentage of IFNγ+ CD8+ lymphocytes of the negative control) was subtracted from the experimental wells (percentage of IFNγ+ CD8+ lymphocytes in the test well) to obtain the antigen-specific CD8+ T-cell frequencies. For the second and third phases, the percentages of specific cytokine-secreting cells were calculated in the CD3+ CD4-negative subset. Gating results, provided in a ppt or pdf file, were subjected to a visual inspection. In the second ICS panel, the acquired events in the FCS files were also centrally analyzed by a single operator at LUMC [13] and re-assessed independently by a second experienced evaluator. A positive response against FLU or CMV was pre-defined as at least twice the background staining and with a clearly visible population of events. Subgroup analysis was performed only when both arms comprised almost equal numbers of participating laboratories to allow for statistical testing (i.e., by Mann–Whitney test). Intercenter variability was evaluated by calculating the coefficients of variation (% CV = SD/mean × 100 %).

### Laboratory environment

The pre-screening by IFNγ-ELISPOT was performed in a central laboratory that does not operate under GLP, following SOPs and using trained staff. The central laboratory has participated in all CIP proficiency panels (http://www.cimt.eu/workgroups/cip/), as well as in IFNγ-ELISPOT panels of the Cancer Immunotherapy Consortium [17, 18], to validate its SOPs. Participating laboratories followed their own established protocol for the ICS assay with previous experience ranging between one and 15 years.

## Results

### Phase 1 proficiency panel for ICS

**Fig. 1:**
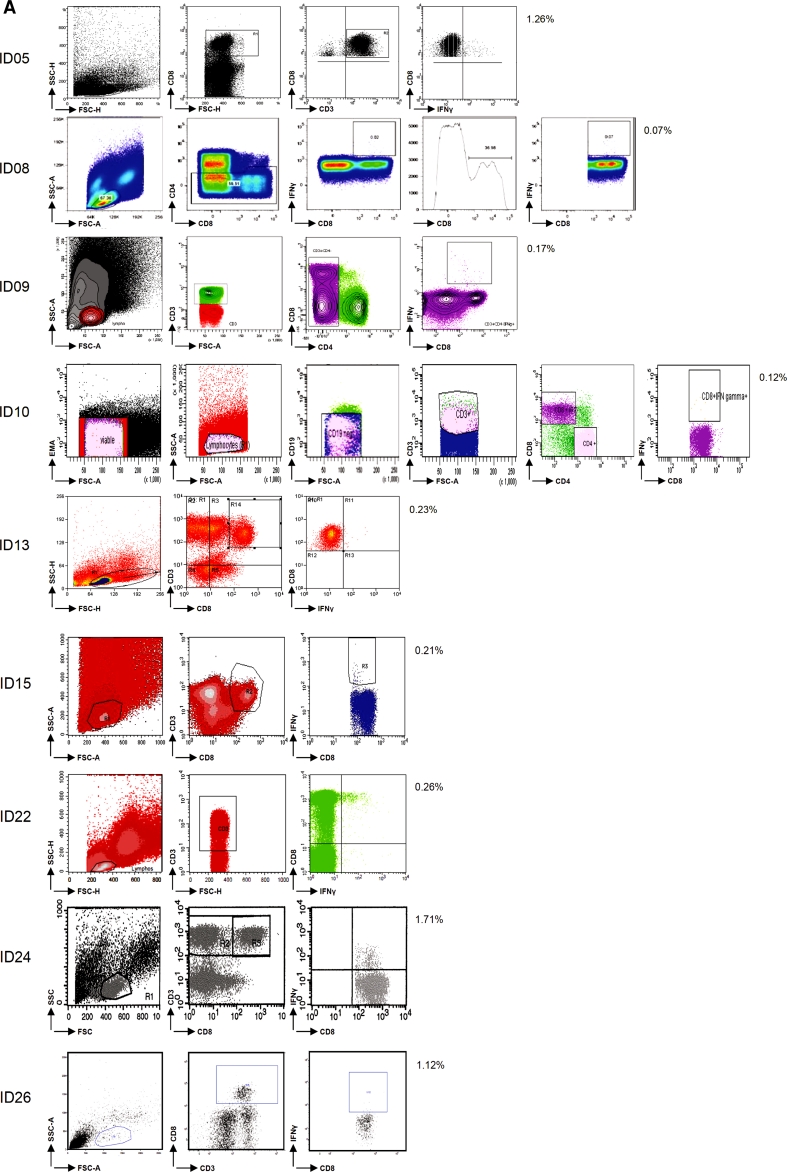

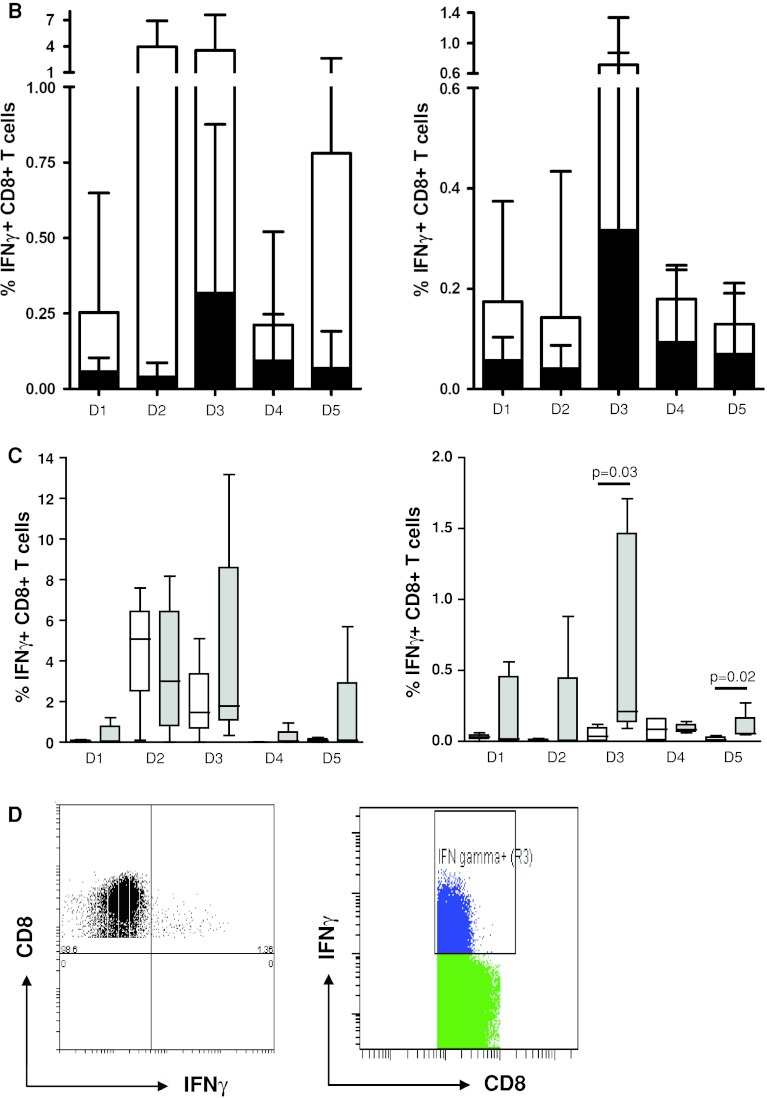
Assay variables influencing the test performance in phase 1. **a** Analysis plots of the same sample (i.e., donor 3 tested for FLU) are shown for all 9 participants as indicated by ID number, demonstrating the variety in frequencies of IFNγ-producing CD8+ T cells and in the gating strategies used. **b** The average frequency (+SD; 9 laboratories) of IFNγ-producing CD8+ T cells is depicted for each donor (*D*) in the negative control samples (*black bars*) and after stimulation with the CMV (*left*) or FLU (*right*) peptides (*white bars*). **c** The frequency of IFNγ-producing CD8+ T cells after stimulation with CMV (*left*) or FLU (*right*) is plotted according to the use of IMDM (4 participants, *white boxes*) or other media (5 participants, *gray boxes*) in the ICS assay for all 5 donors (*D*). Shown are the median, interquartile range and SD. Significant differences as determined by Mann–Whitney test are depicted. **d** Examples of the gating strategy applied for the detection of the IFNγ-producing CD8+ T cells. Some participants did not analyze the whole IFNγ-producing population, but gated through the positive cell population

**Fig. 2:**
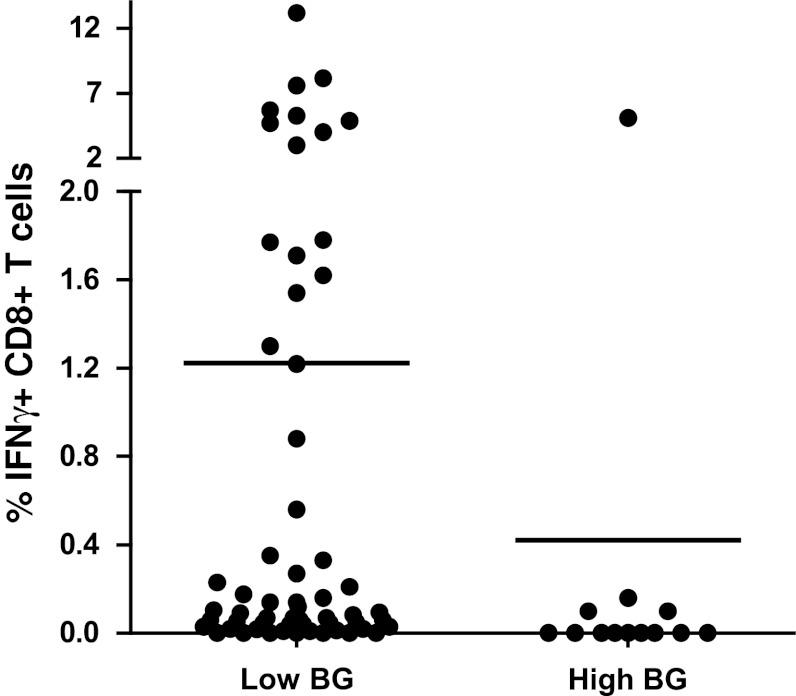
High background staining decreased ability to detect responses in phase 1. The detection (i.e., frequency of IFNγ-producing specific CD8+ T cells after FLU or CMV peptide stimulation) is shown only for the positive donor–antigen combinations versus a low or high background in the corresponding negative control sample. The background was classified as low (*n* = 59) or high (*n* = 13) based on the average background value (0.118 %) measured in all negative control samples (*n* = 72) accumulated from all participants

In this first step, the participants (*n* = 9) used their own ICS protocol for the detection of antigen-specific IFNγ-producing CD8+ T cells (i.e., against FLU and CMV) in the 5 pre-selected donors and reported the responses detected (Online resource 2). Seven participants used unstimulated PBMC (medium only) as a negative control sample. One laboratory used isotype antibody staining and another laboratory fluorescence minus one (FMO, staining of the cells with all antibodies except anti-IFNγ) for the determination of background staining. Of note, in all 3 panel phases, each participant applied the same gate settings within one donor. Most of the participants also applied the same gate settings to all the donors tested in one experiment. Some participants used different gate settings between donors in order to optimize the signal-to-background ratio. However, this did not translate into better or worse capacity to detect a response. Only 1 out of 9 laboratories detected all 8 responses and 3 laboratories detected 6/8 or 7/8 responses (Table 1, phase 1). The frequencies of IFNγ-positive cells reported—after subtraction of the negative control sample value—varied enormously within the group. CV values ranged from 69 to 285 % for the individual donors (Online resource 4B). Exemplary individual plots and the gating strategy are shown for D3 tested with the influenza peptide (FLU) for all 9 laboratories participating in this first phase (Fig. 1a). For each donor, the frequency of IFNγ-positive CD8+ T cells in the control and peptide-stimulated samples is shown in Fig. 1b as an average of all laboratories. Wide variability in the detection of the lower frequency events was the major contributor to the large standard deviation (SD). The frequencies of antigen-specific T cells ranged from at least a 14-fold difference for D5 against FLU (CV = 136 %) up to a 142-fold difference in frequency for D5 against CMV (CV = 262 %). We further observed substantial variability in the background staining within the same donor between the different laboratories and also between the 5 donors tested by the same participating laboratory. As background was subtracted from the positive events, high background increased the risk of missing a response. The detection of responses (*n* = 8; 3× CMV and 5× FLU) was significantly better when the background was below the average background value (0.118 %) of the whole group (*p* = 0.003; Fig. 2).

**Table 1:** Performance per participant and testing phase

Laboratory ID	Phase 1	Phase 2	Phase 2 central analysis
5	8	n.t.	n.t.
8	7	5	3
9	7	8	8
10	6	6	6
13	5	n.t.	n.t.
15	5	7	5
22	2	7	5
24	3	3	3
26	0	n.t.	n.t.
Total to be detected	8	8	8

The performance of each participant is depicted for the ICS proficiency panel phases 1 and 2 (and after central analysis of the phase 2 FCS files). The numbers indicate the number of positive T-cell responses out of a total of 8 reactivities. *n.t.* is not tested as this laboratory was not participating in the indicated phase

In order to identify critical parameters responsible for variability, we then focused on the differences in the assay protocols used by the participating laboratories. A total of 18 different parameters were collected and analyzed (Online resource 3A and 4A), and for some parameters, we were able to stratify results into 2 subgroups containing similar number of laboratories per arm. The activation time (i.e., 5–6 h vs. overnight accumulation of IFNγ) did not influence performance. There was extensive variability in the number of cells recovered after thawing but this did not affect the assay performance as most laboratories used 1–2 million viable cells per donor–antigen combination, which may have compensated for initial cell loss. It appears that the medium (IMDM in 4 laboratories vs. another medium in 5 laboratories) influences outcome and specifically IMDM appeared to reduce the frequency of antigen-specific T cells detected (Fig. 1c).

Central review of the flow cytometry plots revealed a high diversity in the gating strategies used by the participants (Fig. 1a). A surprising observation was that some laboratories missed part of the IFNγ-producing population due to a tight gating on the CD8-high population (Fig. 1d).

### Phase 2 proficiency panel for ICS

**Fig. 3:**
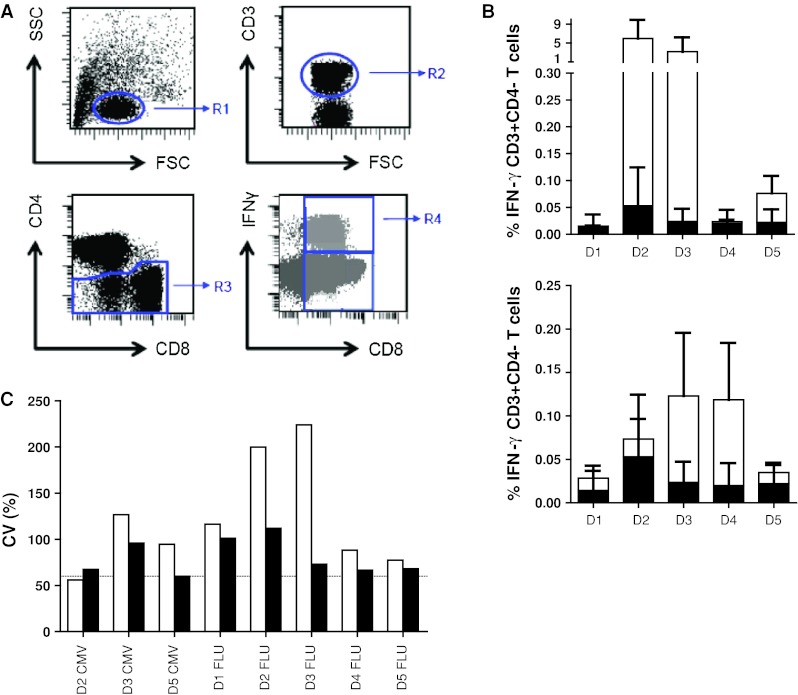
Guidelines partially harmonized ICS performance in phase 2. **a** The gating instructions provided to the participants for the second-phase ICS proficiency panel. First, the lymphocytes are gated (R1), then the CD3 population (R2), followed by plotting CD4 versus CD8 to gate on the CD8 (including the CD8dim) cells (R3), which is called the CD3+ CD4− cell population, and finally the IFNγ-producing CD3+ CD4− T cells can be gated. **b** The average frequency (+SD) of IFNγ-producing CD3+ CD4− T cells is depicted for each donor (*D*) in the negative control samples (*black bars*) and after stimulation with the CMV (*top*) or FLU (*bottom*) peptide (*white bars*). **c** The CV values per donor (*D*)–antigen (CMV or FLU) combination are depicted for those 6 laboratories participating in both phases 1 (*white bars*) and 2 (*black bars*)

A guideline was provided for this second step with three mandatory requirements (Online resource 2). All 6 participants had to use the same X-Vivo 15 medium, following the results from our previous IFNγ-ELISPOT panels [12, 18]. Laboratories had to stain for CD3, CD4 and CD8 and to gate on the CD3+ CD4-negative cell population to include the CD8dim population (Fig. 3a). The third requirement was to use non-stimulated PBMC as a negative control sample. The laboratories were asked to use the second PBMC vial of each of the 5 donors (Table 1, phase 2; Online resource 3B and 4A). The overall results for all laboratories per donor–antigen combination are shown in Fig. 3b. Harmonization resulted in a substantial decrease in variability for some donor–antigen combinations for those laboratories participating in both panels (Fig. 3c, Online resource 4B and 4C); however, the CV values still remained high and above 60 %. Central analysis of the participants' individual FCS files did not result in an increased detection rate or lower CV values, indicating that the gating strategy is not the only parameter of substantial influence on assay outcome (Table 1 and data not shown). The relatively small number of participating laboratories did not allow us to further characterize the protocol parameters responsible for this variability, and larger panels are needed to address this question.

### Phase 3 in silico proficiency panel to harmonize the gating strategy

**Fig. 4:**
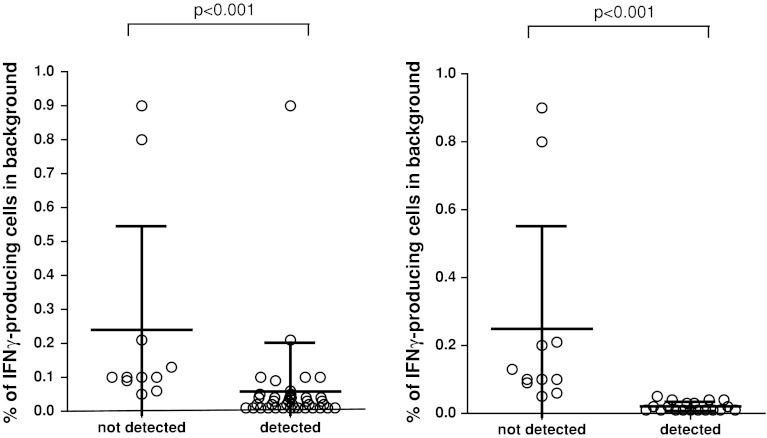
High background staining correlates with a low detection rate in phase 3. The % of IFNγ-producing cells in the negative control sample (i.e., background) versus the detection of a response in the corresponding stimulated sample is depicted for all donor–antigen combinations (and all participants) in which a positive response should have been detected (*left*; *n* = 50 stainings) or in the case that low-frequency responses (as observed against FLU; *n* = 30 stainings) should have been detected (*right*). The background was significantly lower in those donor–antigen combinations where a response was detected

**Fig. 5:**
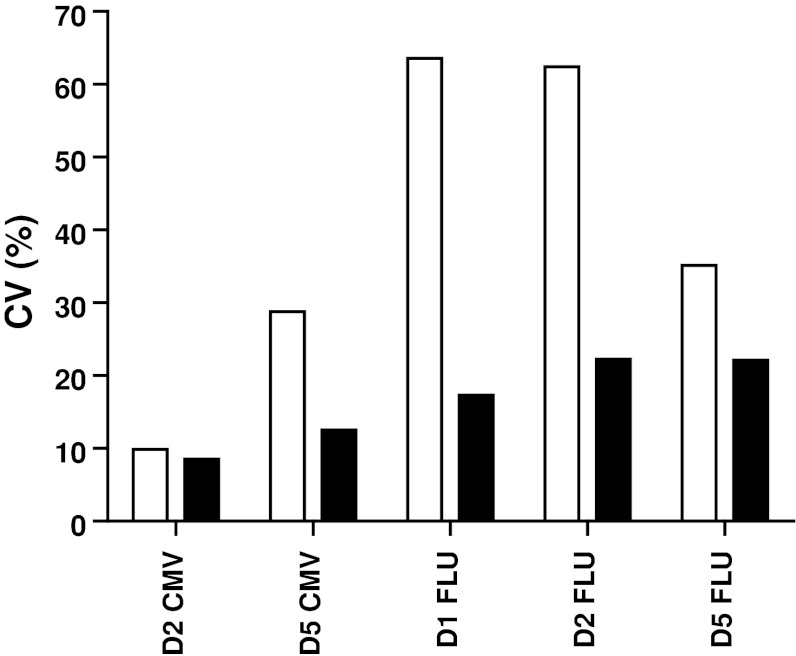
Harmonization of the gating strategy results in acceptable low variation between laboratories participating in phase 3. The coefficient of variation (CV) value is given for the initial analysis of the in silico gating ICS panel (*white bars*) and after instruction of three participants, who then performed a re-analysis (*black bars*) only for the positive reactive donor (*D*)-antigen (CMV or FLU) combination. The CV values dropped after harmonization of the gating strategy

In order to eliminate the role of the wet laboratory and to be able to focus on the impact of gating and data analysis, we undertook a multicentre in silico panel (Online resource 2). All 10 participants received the FCS files of 3 donors (D1, D2 and D5) generated by one of the participating laboratories during the second ICS proficiency panel with one high (≥1 %), one intermediate (≥0.1  and <1 %) and 3 low (<0.1 %) frequency reactivities observed by ICS and included the non-stimulated (medium) and stimulated PBMC samples. Participants analyzed the FCS files according to the same gating strategy that was mandated during the second testing phase (Online resource 4A). All but one participant followed the gating instructions. We found that different approaches were used before the lymphocyte population was gated, in particular exclusion of the doublets or time versus count plot. All 10 laboratories plotted FSC versus SSC to gate on the lymphocytes. Subsequent gating on the CD3+ T cells varied from using histograms or two-dimensional dot plots (CD3 vs. CD4 or CD3 vs. FSC). Following the CD3 selection, 6 laboratories plotted CD4 versus CD8 to gate on the CD3+ CD4− population, whereas three laboratories plotted the CD3+ population in CD3 versus CD4. Then, all laboratories plotted the CD3+ CD4-negative population against IFNγ. Despite the set gating instructions, the measurement of the IFNγ-positive cells varied between the laboratories (Online resource 5A). Central visual analysis of all dot plots revealed that (1) the CD8dim population was still not completely included by 4 laboratories and (2) some of the participants used very tight gates close to the IFNγ-negative cell population, thereby increasing the background staining in the medium control sample. This affected sensitivity, as the data in Fig. 4 demonstrate that the signal-to-noise ratio decreased and this is reflected in an inability to detect low-frequency responses (*p* < 0.001). To confirm this observation, participants using a sub-optimal gating strategy were asked to re-analyze the same FCS files with improved gating. This re-analysis allowed all three laboratories to detect (most of the) low-frequency responses against the influenza peptide with a decrease in the CV values for all donor–antigen combinations below 30 % (Fig. 5; Online resource 5B). The most common findings/errors and recommendations for gating are given in Table 2. In conclusion, this in silico ICS gating panel demonstrated that part of the huge variation in the detection rates and in the frequencies of cytokine-producing T cells between different laboratories is generated at the level of the analysis. We conclude that the gating strategies must be harmonized first for any attempt at identifying wet laboratory contributors on ICS outcome to be successful.

**Table 2:** Common findings/errors and recommendations for gating of ICS data

Findings/errors	Recommendation and reason
1. Gating was done only on CD3−, CD4− and/or CD8− high expressing cells	Include dim populations as these may contain cytokine-producing cells (cells can downregulate co-receptor molecules upon activation)
2. Gate for cytokine-positive cells was set through the cytokine-positive population	Include plots in your gating strategy for CD3+ CD4− (in case of looking at cytokine-producing CD8+ T cells) or CD3+ CD8− (for CD4) T cells to oversee all cytokine-producing cells and be able to gate on the complete cytokine-positive population for that certain T-cell type (so including dim)
3. Gate for cytokine-positive cells was set too close to or included cytokine-negative cells	Set the gate far enough (but not too far) from the non-responding population to decrease the background staining in the unstimulated sample and increase the signal-to-noise ratio

## Discussion

Multiparameter flow cytometry such as the intracellular cytokine staining assay allows the simultaneous assessment of multiple facets of the immune response against a certain antigen. Even more than surface staining, the ICS assay comprises a number of steps that all bear potential influence on the data produced: these include medium, stimulation protocol, staining protocol, instrumental set up, use of negative and positive control samples, number of cells for staining and acquisition, and not the least strategies applied to analyze the results by computer software. Several groups have already reported parameters that might be important for the sensitivity of the intracellular staining assay and have proposed standardized protocols [8, 9, 19–25]. However to date, no harmonization has taken place that allows interlaboratory comparison.

In an effort to harmonize ICS, the CIP conducted three consecutive proficiency panels. In phases 1 and 2, we identified that the choice of test medium and the level of background staining influenced the test performance, in line with previous observations for the ELISPOT assay in our proficiency panels [11–13]. Harmonization of these parameters partly resulted in a decrease in interlaboratory CV values. Moreover, evaluation of these 2 phases suggested that for ICS, a critical aspect is the choice of the gating strategy. We chose to assess this by eliminating the influence of the wet laboratory element of the assay and undertook an in silico panel, where participants were asked to analyze previously acquired data from one laboratory. This successfully allowed us to harmonize the gating strategy and resulted in a CV value below 30 %. The most important steps were (1) the inclusion of the CD8dim population, which contains many IFNγ-producing CD8+ T cells following activation-mediated downregulation of CD8 and (2) setting the gate wide enough from the non-responding population to optimize the signal-to-noise ratio. We conclude that harmonization of gating strategies is the first requirement before other parameters that influence assay outcome can possibly be identified.

Although it is successful for harmonizing the IFNγ-ELISPOT assay and the flow cytometric-based HLA multimer assay [13], a simple 2-step method was not sufficient to harmonize the ICS assay. Central analyses of the datasets provided by the participants did not substantially decrease the CV values indicating that in addition to the gating strategy, there are protocol-related variables that would benefit from harmonization. The group of participants was too small to identify statistically significant effects, but this will be addressed in following CIP panels. Indeed, for ELISPOT harmonization, we have previously overcome this by the inclusion of a larger number of participants; this could be combined with the use of one standard operation procedure (SOP) in which systematically one variable is tested by all participants. In the field of HIV immune research, the analysis of specific T cells by ICS is fully standardized by providing a SOP as well as the peptides and lyophilized antibodies prefilled in 96-well plates [7, 8, 26–28]. However, this is more difficult and less likely to be feasible in the field of immunotherapy of cancer where the antigens of interest vary between different cancer types and between the many laboratories involved. Nevertheless, recommendations for the gating strategy can be given and are listed in Table 2, which were demonstrated to provide some harmonization in this stage of the ICS assay. Following these gating guidelines will give the opportunity to be able to study and identify parameters in the wet laboratory protocol influencing test performance.

### Electronic supplementary material

Below is the link to the electronic supplementary material.

Supplementary material 1 (PDF 4488 kb)

## Appendix

Participants of one or more ICS proficiency panel(s) of the CIP:

1. E. Kämpgen, M. Wiesinger, S. Gross. Department of Dermatology, University Hospital, Erlangen, Erlangen, Germany.
2. G. Pawelec, E. Derhovanessian. Tübingen Ageing and Tumour Immunology Group, Center for Medical Research, University of Tübingen, Tübingen, Germany.
3. B. Seliger, D. Riemann. Institute of Medical Immunology, Martin Luther University, Halle, Germany.
4. C. Britten, S. Attig. Mainz, Department of Internal Medicine III, Johannes Gutenberg University, Mainz, Germany.
5. K. Giannopoulos. Department of Experimental Hematooncology, Medical University of Lublin, Lublin, Poland.
6. H. Pohla. Laboratory of Tumour Immunology, Ludwig-Maximilians-University, Munich, Germany.
7. M. Schmitt, A. Schmitt. Department of Cellular Immunotherapy, University Clinic Heidelberg, Heidelberg, Germany.
8. C. Gouttefangeas, S. Attig, K. Laske. Department of Immunology, Interfaculty Institute for Cell Biology, Eberhard Karls University, Tübingen, Germany.
9. S. H. van der Burg, M.J.P. Welters. Department of Clinical Oncology, Leiden University Medical Center, Leiden, The Netherlands.
10. C. Ottensmeier, A. Mander, A. Cazaly. Cancer Sciences Division, University Hospitals, Southampton, UK.
11. A. Paschen, F. Zhao. Department of Dermatology, University Hospital Essen, Essen, Germany.
12. A. Letsch. Department of Hematology, Oncology, Charité Campus Benjamin Franklin, Berlin, Germany. C. Scheibenbogen. Department of Medical Immunology, Charité Campus Virchow-Klinikum, Berlin, Germany.
13. F. Kern. Division of Medicine, Brighton and Sussex Medical School, Brighton, UK.
