# Supplementary material for: Harmonization of the intracellular cytokine staining assay
Source: Cancer Immunol Immunother. 2012 May 22;61(7):967–78. doi: 10.1007/s00262-012-1282-9 (PMC3378841; doi:10.1007/s00262-012-1282-9)
Supplement: Supplementary file 1 — Supplementary material 1 (PDF 4488 kb) [file 262_2012_1282_MOESM1_ESM.pdf]

A

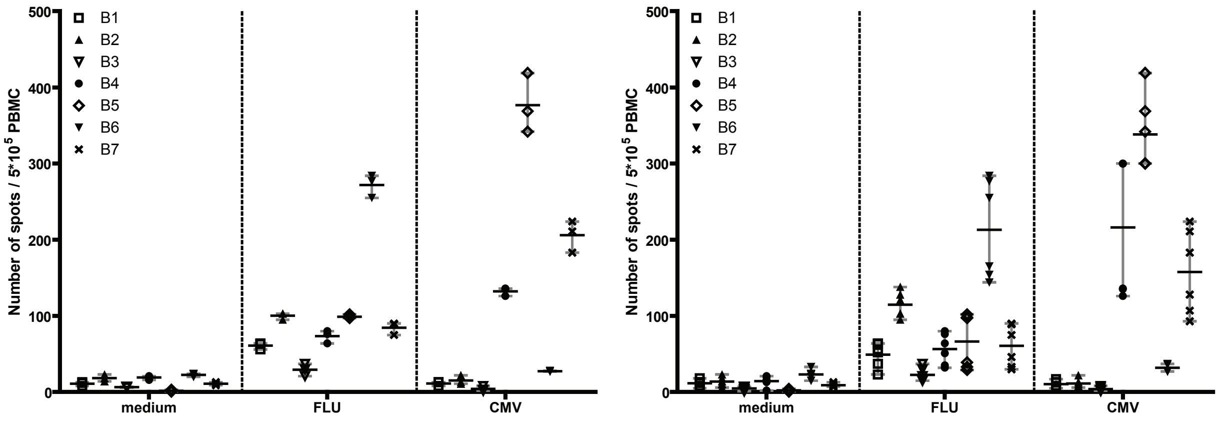

B

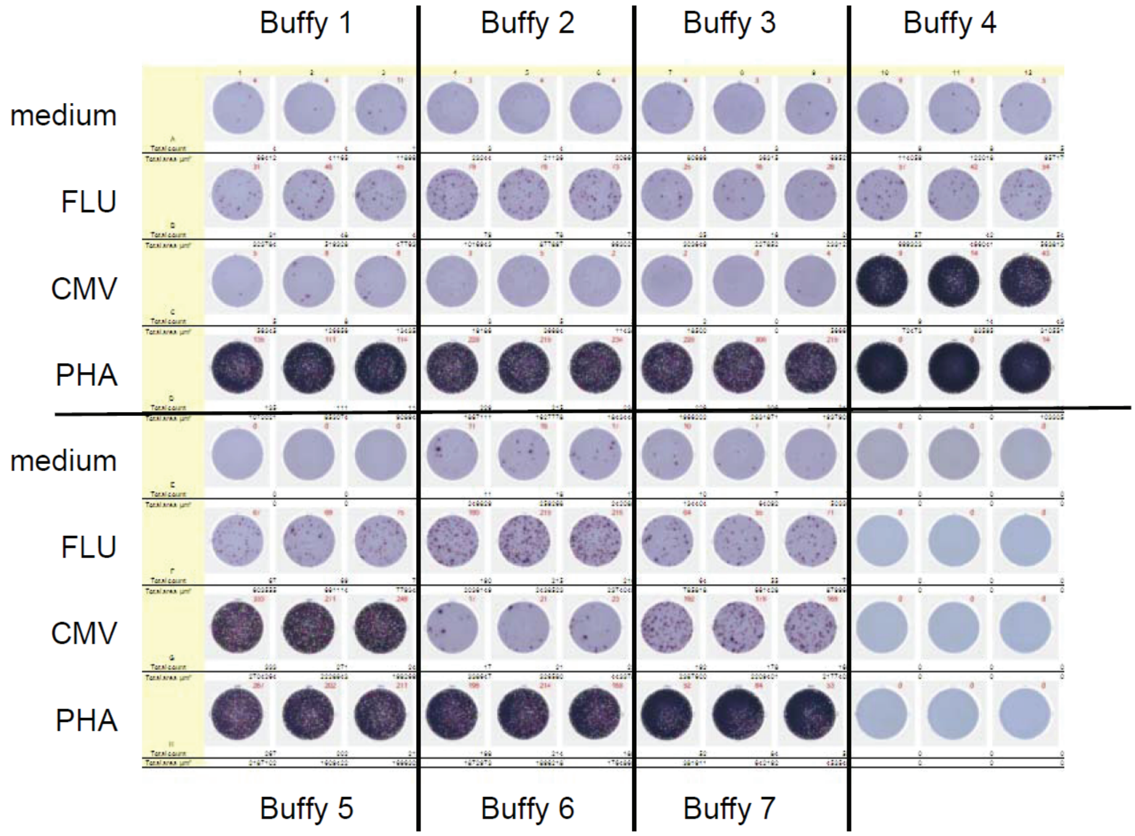

Online resource 1: Pre-screening of donors by IFNγ-ELISPOT

(A) The IFNγ-ELISPOT results are depicted. Left graph shows the first screening test (in triplicate wells) and the right graph the combined data of this first and a second independent screening test. PBMC isolated from 7 buffy coats (B) were analyzed for their recognition of the model antigens influenza M1 (FLU) or human pp65 CMV (CMV). **(B)** As an example the IFNγ-ELISPOT plate of the first pre-test is shown. PHA was taken along as a positive control.

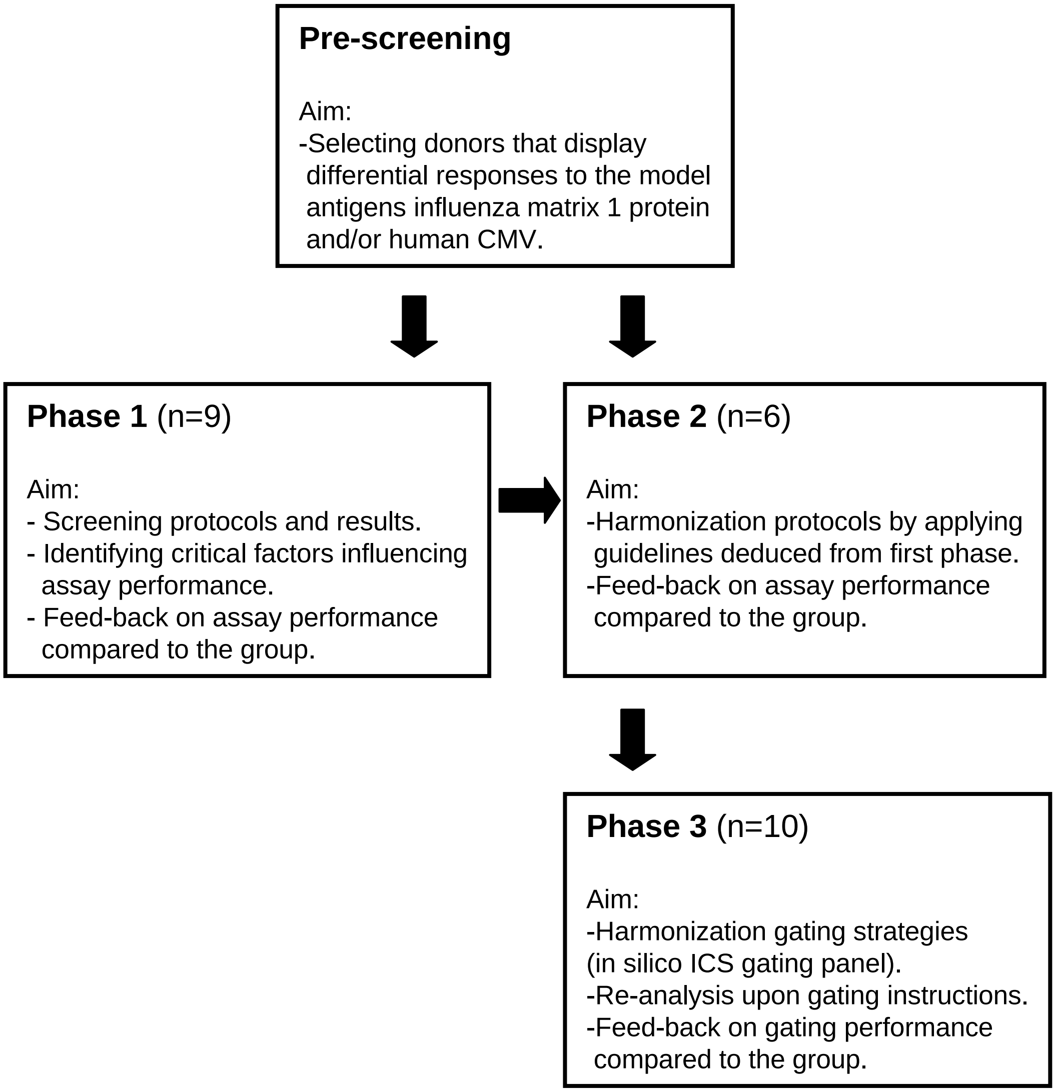

Online resource 2: **Schematic overview of the three consecutive proficiency panels**

A

ID	Resting time (hours)	Number of cells per test	Peptide conc. (µg/ml)	Medium	Serum	CD8 antibody Clone	Fluor chrome	Company	Dilution	CD3 antibody Clone	Fluor chrome	Company	Dilution
5	16	2 x 10e6	2	RPMI	human AB	SK1	PerCP	BD	1:5	UCHT1	PE	BD	1:5
8	16	2 x 10e6	5	IMDM	human AB	SFCI21 ThyD3	PE-Cy7	BC	1:120	13B8.2#	APC	BC	1:75
9	26	1-2 x 10e6	5	IMDM	human AB	SK1	APC-Cy7	BD	1:50	UCHT1	Pe-Cy7	BD	1:50
10	0	2 x 10e6	5	X-VIVO	none	SK1	APC-H7	BD	1:16	UCHT1	PE	Caltag	1:25
13	2	1 x 10e6	1	RPMI	human AB	SK1	PerCP	BD	1:10	UCHT1	PE	BD	1:10
15	18	1 x 10e6	5	RPMI	FCS	SK1	FITC	BD	1:50	SK7	PerCP-Cy5	BD	1:50
22	16	2 x 10e6	5	IMDM	human AB	SK1	APC	BD	1:10	SK7	PerCP	BD	1:5
24	2	0.25 x 10e6	5	RPMI	FCS	BW 135/80	APC	Milthenyi	1:20	UCHT1	PE	BC	1:20
26	16	0.7-1.7 x 10e6	2	IMDM	human AB	SK1	PerCP	BD	1:2.5	UCHT1	FITC	BC	1:5

ID	IFNg antibody Clone	Fluor chrome	Company	Dilution	Secretion blocking reagent (final conc.)	Addition of blocking reagent after	Activation time	Lyse/fix reagent	Permealization reagent	Duration of IFNg staining
5	4S.B3	FITC	BD	1:25	Golgi-Stop (1 µl/ml)	1 hour	overnight	Fix (Dako)	Perm (Dako)	15 min at RT
8	B27	FITC	BD	1:200	Brefeldin A (50 µg/ml)	Immediately	5 hours	Fix (BD)	Perm (BD)	30 min at 4°C
9	4S.B3	FITC	BD	1:50	Brefeldin A (10 µg/ml)	1 hour	overnight	4% paraphormaldehyde	saponine	30 min at 4°C
10	B27	PE-Cy7	BD	1:10	Golgi-Plug (1 µg/ml)	1.5 hours	overnight	Fix (BD)	Perm (BD)	30 min at 4°C
13	4S.B3	FITC	BD	1:50	Golgi-Stop (1 µl/ml)	1 hour	overnight	Fix (Dako)	Perm (Dako)	15 min at RT
15	B27	APC	BD	n.a.	Brefeldin A (10 µg/ml)	1 hour	6 hours	1% Formaldehyd-2mM EDTA	saponine	20 min at 4°C
22	25723.11	FITC	BD	1:2.5	Brefeldin A (7.5 µg/ml)	1 hour	6 hours	Lyse (BD)	Perm (BD)	30 min at 4°C
24	25723.11	FITC	BD	1:10	Brefeldin A (10 µg/ml)	2 hours	6 hours	Fix (An der Grub)	Perm (An der Grub)	15 min at 4°C
26	45-15	APC	IQP	1:50	Brefeldin A (n.a.)	2 hours	6 hours	Lyse (BD)	Perm (BD)	30 min at 4°C

B

ID	Resting time (hours)	Number of cells per test	Peptide conc. (µg/ml)	Medium*	Serum*	CD4 antibody Clone	Fluor chrome	Company	Dilution	CD3 antibody Clone	Fluor chrome	Company	Dilution
8	16	2 x 10e6	5	X-VIVO	none	13B8.2	PE	BC	1:75	S4.1	APC	Caltag	1:75
9	24	2 x 10e6	5	X-VIVO	none	SK3	Pe-Cy7	BD	1:50	UCHT1	PB	DAKO	1:20
10	0	2 x 10e6	10	X-VIVO	none	RPA-T4	PB	Biolegend	1:250	UCHT1	PE	Caltag	1:50
15	8	5 x 10e6	5	X-VIVO	none	RPA-T4	Fitc	BD	1:10	SK7	PercP	BD	1:10
22	24	3 x 10e6	5	X-VIVO	none	SK3	APC	BD	1:16	SK7	PercP	BD	1:5
24	1	1x 10e6	2	X-VIVO	none	SK3	PercP	BD	1:10	UCHT1	Fitc	BD	1:10

ID	IFNg antibody Clone	Fluor chrome	Company	Dilution	Secretion blocking reagent (final concentration)	Addition of blocking reagent after	Activation time	Lyse/fix reagent	Permealization reagent	Duration of IFNg staining
8	B27	FITC	BD	1:200	Brefeldin A (10 µg/ml)	1 hour	6 hours	Cytofix/Cytoperm (BD)	saponine	30 min at 4°C
9	4S.B3	FITC	BD	1:50	brefeldin A (10 µg/ml)	1 hour	18 hours	4% paraphormaldehyde	saponine	30 min at 4°C
10	B27	PE-Cy7	BD	1:20	GolgiPlug (1 µg/ml)	Immediately	overnight	Cytofix/Cytoperm (BD)	Perm/Wash (BD)	30 min at 4°C
15	25723.11	APC	BD	1:33	Brefeldin A (5 µg/ml)	Immediately	12 hours	Permwash (eBioscience)	eBioscience	25 min at 4°C
22	25723.11	FITC	BD	1:2.5	Brefeldin A (7.5 µg/ml)	2 hours	18 hours	Lyse (BD)	Perm (BD)	20 min at 4°C
24	25723.11	PE	BD	1:5	Brefeldin A (10 µg/ml)	2 hours	6 hours	Lyse (BD)	Perm (An der Grub)	15 min at 4°C

Online resource 3: ICS protocol variables

The variables in the wet protocol of the ICS as conducted in proficiency panels 1 **(A)** and 2 **(B)** are given. * indicates the obligatory recommendations; # not a CD3 but CD4 antibody was used; BD, Becton Dickenson; BC, Beckman Coulter; PC, pacific blue; n.a. information not available.

Phase	Parameter	Average	Median	Range	CV (%)
1	**Number of viable cells (directly after thawing)**	$15.5x10^6$	$15.1\ x10^6$	$4.9 - 39.7\ x10^6$	43.9
	**% Dead cells (directly after thawing)**	16.0	9.3	3.6 - 53.3	87.7
	**Number of viable cells (after resting)**	$11.4\ x10^6$	$10.6\ x10^6$	$2.7 - 34.2\ x10^6$	61.1
	**% Dead cells (after resting)**	24.6	10.7	1.5 - 82.0	97.0
	**Number of lymphocytes**	278521	163873	9198-1229921	114
	**Number of CD8+ T-cells**	63105	25703	623-335556	131
	**% CD8+ T-cells**	21.6	20.8	7.4-35.6	36.9
2	**Number of lymphocytes**	675243	715001	199922-1349327	33.7
	**Number of CD3+ T-cells**	591350	556426	143889-1200000	41.3
	**% CD3+ T-cells**	78.4	79.0	57.7-93.0	9.9
	**Number of CD3+CD4- T-cells**	204433	191131	52234-495797	49.4
	**% CD3+CD4- T-cells**	34.0	32.3	19.1-47.0	19.1
3	**Number of lymphocytes**	507956	505967	411364-673447	10.3
	**Number of CD3+ T-cells**	418646	419665	319571-559130	11.0
	**% CD3+ T-cells**	82.4	83.8	76.1-85.9	3.6
	**Number of CD3+CD4- T-cells**	160623	164364	85598-263390	26.4
	**% CD3+CD4- T-cells**	38.3	43.5	22.1-48.5	22.6

Online resource 4A: Parameters in the 3 phases of the ICS proficiency panels.

CMV					
**ID**	**D1**	**D2**	**D3**	**D4**	**D5**
**5**	0.360	**3.000**	**1.780**	0.060	**0.140**
**8**	0.010	**7.590**	**1.620**	0.010	**0.070**
**9**	0.060	**4.870**	**1.300**	0.000	**0.230**
**10**	0.000	**4.696**	**1.771**	0.013	**0.105**
**13**	1.205	**8.169**	**13.17**	0.941	0.046
**15**	0.000	0.000	**0.330**	0.050	**5.680**
**22**	0.000	**5.280**	**5.090**	0.000	0.000
**24**	0.000	**1.540**	**4.020**	0.000	0.040
**26**	0.128	0.100	0.000	0.000	0.100
**Median**	0.010	4.696	1.771	0.010	0.100
**Average**	0.196	3.916	3.231	0.119	0.712
**SD**	0.396	2.986	4.066	0.309	1.864
**CV**	202	76.3	126	259	262

FLU					
**ID**	**D1**	**D2**	**D3**	**D4**	**D5**
**5**	**0.560**	**0.880**	**1.220**	**0.140**	**0.270**
**8**	**0.030**	0.010	**0.070**	**0.160**	**0.040**
**9**	**0.060**	**0.020**	**0.120**	0.010	**0.020**
**10**	0.017	0.012	**0.091**	**0.095**	**0.054**
**13**	**0.352**	0.000	**0.176**	**0.083**	0.046
**15**	0.000	0.000	**0.210**	**0.070**	**0.050**
**22**	0.030	0.000	0.000	0.000	0.000
**24**	0.000	0.000	**1.710**	0.060	0.060
**26**	0.000	0.000	0.000	0.160	0.000
**median**	0.030	0.000	0.120	0.083	0.046
**average**	0.117	0.102	0.400	0.086	0.060
**SD**	0.200	0.292	0.620	0.059	0.082
**CV**	172	285	155	68.5	136

Online resource 4B: Percentage of IFNγ-producing CD3+CD8+ T cells as reported by participants in Phase 1 of ICS panel.

In bold the positive responses (i.e. at least twice above the negative control with a clearly visible population of events). The values are the percentages of IFNγ+CD3+CD8+ T cells after subtraction of the negative control value. The gray boxes indicate the donor (D)-antigen (CMV or FLU) combinations which were found to be negative in the pre-screening test.

CMV					
**ID**	**D1**	**D2**	**D3**	**D4**	**D5**
**8**	0.000	**4.810**	**1.040**	0.010	**0.030**
**9**	0.003	**8.727**	**3.623**	0.002	**0.080**
**10**	0.000	**4.753**	**0.846**	0.007	**0.049**
**15**	0.002	**12.40**	**8.505**	0.001	**0.107**
**22**	0.000	**1.827**	**4.102**	0.000	**0.029**
**24**	0.000	**2.820**	**0.730**	0.000	0.030
**Median**	0.000	4.781	2.332	0.002	0.040
**Average**	0.001	5.889	3.141	0.003	0.054
**SD**	0.001	3.969	3.014	0.004	0.033
**CV**	165	67.4	96.0	126	60.0

FLU					
**ID**	**D1**	**D2**	**D3**	**D4**	**D5**
**8**	0.000	0.000	**0.010**	**0.060**	0.000
**9**	**0.015**	**0.027**	**0.115**	**0.133**	**0.027**
**10**	0.001	0.000	**0.025**	**0.091**	**0.012**
**15**	**0.033**	**0.026**	**0.145**	**0.209**	0.015
**22**	0.006	**0.061**	**0.202**	**0.079**	**0.013**
**24**	0.030	0.010	**0.100**	0.020	0.010
**median**	0.011	0.018	0.108	0.085	0.012
**average**	0.014	0.021	0.099	0.099	0.013
**SD**	0.014	0.023	0.073	0.066	0.009
**CV**	101	112	73.1	66.5	67.9

Online resource 4C: Percentage of IFNγ-producing CD3+CD4-negative T cells as reported by participants in Phase 2 of ICS panel.

In bold the positive responses (i.e. at least twice above the negative control with a clearly visible population of events). The values are the percentages of IFNγ+CD3+CD4-negative T cells after subtraction of the negative control value. The gray boxes indicate the donor (D)-antigen (CMV or FLU) combinations which were found to be negative in the pre-screening test.

Lab ID	Medium			CMV			FLU		
	D1	D2	D5	D1	D2	D5	D1	D2	D5
**8**	0.01	0.02	0.01	0.00	**10.42**	**0.11**	**0.02**	**0.04**	**0.05**
**9**	0.01	0.02	0.01	0.00	**9.52**	**0.10**	**0.03**	**0.03**	**0.04**
**10**	0.01	0.04	0.03	0.00	**11.46**	**0.10**	**0.03**	**0.06**	**0.05**
**11**	0.13	0.21	0.10	0.06	**13.09**	**0.12**	0.01	0.16	0.08
**12**	0.80	0.90	0.04	0.00	**10.91**	**0.08**	0.00	0.12	**0.04**
**14**	0.01	0.02	0.01	0.00	**12.48**	**0.14**	**0.04**	**0.04**	**0.07**
**15**	0.01	0.03	0.01	0.00	**11.67**	**0.10**	**0.03**	**0.04**	**0.05**
**16**	0.01	0.05	0.09	0.00	**12.55**	**0.11**	**0.03**	**0.05**	0.05
**22**	0.05	0.04	0.06	0.00	**12.46**	**0.10**	0.03	**0.05**	0.04
**24**	0.30	0.20	0.10	0.00	**12.80**	**0.20**	0.00	**0.10**	0.10
**Median**	0.010	0.040	0.044	0.00	12.07	0.11	0.030	0.050	0.050
**Average**	0.13	0.15	0.046	0.006	11.73	0.12	0.022	0.069	0.057
**SD**	0.26	0.28	0.040	0.019	1.16	0.033	0.014	0.043	0.020
**CV**	196	185	87.6	316	9.88	28.8	63.6	62.4	35.1

Online resource 5A: Phase 3 results of in silico ICS gating panel.

In bold the positive responses (i.e. at least twice above the negative control). Note that for CMV and FLU the values are shown after subtraction of medium control.

Lab ID	Medium D1	Medium D2	Medium D5	CMV D1	CMV D2	CMV D5	FLU D1	FLU D2	FLU D5
**8**	0.01	0.02	0.01		**10.42**	**0.11**	**0.02**	**0.04**	**0.05**
**9**	0.01	0.02	0.01		**9.52**	**0.10**	**0.03**	**0.03**	**0.04**
**10**	0.01	0.04	0.03		**11.46**	**0.10**	**0.03**	**0.06**	**0.05**
**11**	0.01	0.03	0.07		**12.44**	**0.11**	**0.03**	**0.03**	0.03
**12**	0	0.03	0.02		**11.62**	**0.10**	**0.03**	**0.04**	**0.05**
**14**	0.01	0.02	0.01		**12.48**	**0.14**	**0.04**	**0.04**	**0.07**
**15**	0.01	0.03	0.01		**11.67**	**0.10**	**0.03**	**0.04**	**0.05**
**16**	0.01	0.05	0.09		**12.55**	**0.11**	**0.03**	**0.05**	0.05
**22**	0.05	0.04	0.06		**12.46**	**0.10**	0.03	**0.05**	0.04
**24**	0.01	0.05	0.03		**11.83**	**0.13**	**0.03**	0.03	**0.05**
**Median**	0.010	0.030	0.025		11.75	0.11	0.030	0.040	0.050
**Average**	0.013	0.033	0.034		11.65	0.109	0.030	0.042	0.048
**SD**	0.013	0.012	0.029		0.99	0.014	0.005	0.009	0.011
**CV**	102.8	35.2	85.6		8.54	12.6	17.3	22.3	22.1

Online resource 5B: Re-analysis of in silico ICS gating panel after new gating instructions.

In bold the positive responses (i.e. at least twice above the negative control). Note that for CMV and FLU the values are shown after subtraction of medium control. The gray boxes indicate the 3 labs that performed the re-analysis.
